# Report of the Veterinary Department of the Minnesota State Board of Health for Quarter and Year Ending, January 1, 1898 (Concluded)

**Published:** 1898-06

**Authors:** 


### CONTROL WORK.

### REPORT OF THE VETERINARY DEPARTMENT OF THE MINNESOTA STATE BOARD OF HEALTH FOR QUARTER AND YEAR ENDING JANUARY 1, 1898.

(Concluded from page 188.)

### Glanders-Farcy.

I am pleased to report that the work with glanders-farcy during 1987 was very satisfactory. Number of horses tested with mallein, •391 ; and number killed or died of glanders-farcy under observation, 180.

*Distribution.* There has been a peculiar distribution of the reported outbreaks during the past year. You will notice by referring to the map that the disease has been prevalent in the extreme northwestern portion of the State, involving Marshall and Polk Counties. During the past year 7 horses have been destroyed in Marshall and 34 in Polk. Then there is a strip of territory covering several townships in width, beginning near Willmar, in Kandiyohi County, running southwesterly through the eastern portion of Chippewa into Yellow Medicine County, in which glanders-farcy has been very prevalent during the past season. In this district there have been 8 cases in Kandiyohi County, 22 in Chippewa, 3 in the eastern portion of Lac-qui-parle and 5 in Yellow Medicine. Another quite serious outbreak occurred in Redwood County, involving a loss of 6 horses. In Faribault County, 7 horses ; Winona, 8 ; 11 in the northwestern corner of McLeod ; 30 just over the line in Carver County ; 9 in Hennepin ; Wilkin, 13, with more cases which are probably affected with glanders-farcy, under quarantine, and 7 horses to be tested with mallein. We will undoubtedly find some cases in the latter group.

I would like to call the attention of this Board to the serious prevalence of the disease in the western portion of the State. The disease has appeared in Murray, Lyon, Yellow Medicine, Lac-qui-parle, Big Stone, Wilkin, Blue Earth, and Marshall Counties.

In addition to these there have been less serious outbreaks in Cass, Todd, Crow Wing, Isanti, Stearns, Stevens, Cottonwood, Murray, Waseca, Mower, Lyon, Lac-qui-parle, and Big Stone Counties.

*Explanation of this distribution.* The explanation of this serious prevalence of glanders-farcy in the extreme western portion of the State probably lies in the fact that herds of Western horses are frequently driven across the border into that portion of the State. Many of these horses are sold before the herds are removed to the Eastern markets.

A serious prevalence of glanders-farcy in certain of the interior counties can be explained by the statement that the disease has previously existed in these neighborhoods, and the people traded and moved these infected horses about, thus spreading the infection over the neighborhoods.

I believe that the work done with these interior outbreaks has been very thorough, but others will undoubtedly come to light during 1898.

It is only fair to these counties to explain that this showing is not made because they are necessarily worse infected or the people more careless, but rather that their veterinarians and local health-officers have been active and efficient.

I believe that this State Board of Health should coöperate with the National Bureau of Animal Industry and the proper State officials of North and South Dakota, and perhaps Montana, to protect northwestern counties, and that this should be done in the near future.

*What horses to test and when to kill.* The question has arisen several times during the past year whether it was wise to insist on testing all horses that had been exposed in the stable with undoubted glanders, and to condemn for slaughter all horses which gave mallein-reaction.

Our work during the past year has emphasized the startling fact that there are many cases of glanders-farcy which show no external symptoms of the disease so far as the most expert veterinarian can observe. Work done by Nocard and other veterinarians in Europe, and by Dr. Williams and others in this country, indicates that there are occasional recoveries of mild cases under favorable conditions, and it is this fact that has caused us to do considerable hard thinking on the problems just stated. Under some circumstances it is undoubtedly practical to order partial quarantine for a long period of time, during which one or more mallein-tests may be given with a view to estimating the progress of the disease. Horses that do not react after the second or third injection may, as shown by Nocard, be considered as having recovered from the disease; but this rule may not be practical for general use. We have no means for estimating at time of test whether an animal may recover; neither can we know in any given case that an animal which is not discharging from the nose or from farcy-sores is not infectious. In fact, there is reasonable evidence that they may be infectious. The plan which we have had in operation during the past year is outlined quite fully in the attached copy of stock-letter which is sent to chairmen, and in the accompanying circular concerning glanders-farcy soon to be issued.

*Methods of dealing with glanders-farcy in other States.* This was fully reported by me at our meeting on July 13th, and need not be reported at this time further than to remind you, in considering this question, that the methods differ widely in different States. In some States they use mallein only on horses which show clinical symptoms of the disease, and are therefore suspicious; in other States they test the entire stable when there is reason to suspect the presence of glanders-farcy, and condemn for immediate slaughter all that show clear mallein-reactions, whether the patients show other symptoms of the disease or not. In other States the mallein-test is not used officially at all, but I think it a fair statement that mallein is used extensively in all States where the sanitary work along veterinary lines is most progressive and satisfactory. The experimental work that has been done in this country and abroad during the past few years has proved beyond question that mallein is an accurate diagnostic for glanders. The most important points that remain to be settled are the limit of its use and what horses shall be condemned for immediate destruction. My own views are given in the proposed circular concerning glanders-farcy.

*Relief for owners.* It has occurred several times during the past season that we have felt compelled to work severe hardships upon owners who were unable to bear the losses. I hope that some arrangement may be made in the near future by which we may be able to reimburse in part, and perhaps at option, according to circumstances. I would not expect to do this as a rule, but for the present only in cases where our work resulted in severe losses to owners who were entirely innocent and were willing to do what was best for the public. We have had several instances of this kind during the year, and I assure you they are not agreeable experiences. In some cases we could do great good if we could only assist the owners to purchase one team of horses able to do farm work, and such horses need not be expensive.

*a. Instructions to Local Health-officers Relative to Glanders-farcy.*

Your letter of — received. I send you a complete glanders-file, consisting of the law dealing with infectious diseases of animals, and a number of circulars, each of which explains its intended use. Please fill out and return to me promptly all that are intended for this office.

It is the duty of the township supervisors, who constitute the local Board of Health in the country or the health-officer of a city, to quarantine any horse or horses suspected of having glanders or farcy. Such horses should be quarantined in care and at the expense of the owner, preferably in the stable wherein they have been previously kept.

You will see by reading the law that it is the duty of the local health-officer or local board to employ a competent veterinarian or physician to make such investigations as he may deem necessary before expressing a positive opinion, after which you must take such action as you may deem best, bearing in mind that animals affected with any infectious disease have no value so far as our work is concerned. The mallein-test should be used in all cases where there is a doubt concerning the nature of the disease. All horses which have been exposed by associating in the stable or otherwise with the ones that have farcy or glanders, or that may be reasonably supposed to have the disease, shall be tested. There sometimes appear mild or latent cases, and cases of recent infection that show no external evidence of the disease, and yet will react upon the mallein-test. Such horses are, or probably will become, dangerous.

You have full authority under the law to order the killing and burning or deep burial of the carcasses.

I send you to-day — c.c. mallein, sufficient for testing — horses, and also blanks for recording results of test. I trust the injections will be made and the temperatures taken at the hours and intervals suggested on these blanks, certainly at corresponding intervals. It is your duty to see that the work is carefully done and that all blanks are carefully filled out.

You must act on your own judgment in this matter and not be influenced unduly by what an owner may say or think. Owners are often dissatisfied when farcy or glanders is found in their stables, and often wish to employ some other veterinarian or have something else done that will simply delay action. I would not urge you to be hasty, however; matters like this should be dealt with carefully and thoughtfully.

It is only just to the owner that the necessary examinations be made promptly, and quarantine released as soon as the owner has complied with your instructions in regard to killing, disposal of carcasses, and disinfection of premises.

If there is anything further that the State Board can do to assist in this matter, let me know. Please keep me closely informed as to what you are doing or have done. Very respectfully,

M. H. Reynolds.

*b. Circular of Information for Local Health-officers Concerning Glanders-farcy.*

In all ordinary cases of suspected glanders-farcy, first quarantine the suspected animals, then call in a competent veterinarian, who should make such examinations and tests as he may deem necessary. The further action of the board to be largely determined by the diagnosis and advice of the veterinarian.

All horses must be included in this preliminary quarantine that are discharging from the nose or that have had recent sores upon the body, and all horses that have worked as mates with such horses.

The mallein-test should be conducted as nearly as possible according to the plan given in the blanks which are sent out from this office with the mallein. This plan for the test has been found very convenient, and it is very important for the proper keeping of office-records that the injection should be made and the temperatures taken at the same hours by different veterinarians.

Test all horses that have been exposed in the stable to cases that may be reasonably suspected of having the glanders-farcy.

It is most economical and usually most satisfactory to have the veterinarian who makes the examination and conducts the mallein-test finish up the work at one trip.

All horses that give a clear reaction on mallein-test, and show external symptoms of the disease: all horses which react on mallein-test, and have chronic cough or any chronic lung-trouble, thick wind, etc., and all horses which show positive symptoms of the disease, without a clear mallein-test, must be destroyed without delay. All horses which react, but show no other symptoms of the disease, may either be killed at once, or quarantined to be retested at the end of thirty days, or continued under partial quarantine, for retest, for a period not to exceed one year from first test.

Horses that have reacted twice with an interval of thirty days or more, whether showing other symptoms of the disease or not, must either be killed at once or continued under partial quarantine for a period not longer than a year from first test. If at any time within the year these horses show external symptoms of the disease they must be killed.

If they show no external symptoms of the disease, but react again at the end of the year, they must be continued under partial quarantine until they have either been retested twice, with an interval of thirty days, without reaction and released, or have developed external symptoms of the disease and have been killed. During this time they must not be sold for removal from the place. In case it is decided to retest at the end of thirty days the same ruling must be applied as though the test had occurred at the end of the year.

In all cases where retests are made, the second dose should be one-half larger than the first dose.

It is not always necessary to rigidly quarantine all the horses on a farm, but all cases that are discharging from the nose or from sores upon the body must be rigidly quarantined. The veterinarian can often select a team that will be reasonably safe to allow upon the road. Such a team must not be put in other than the owner's stable, or tied to any public hitching-post or rack, or be watered from any public watering-trough or tank.

Horses affected with glanders-farcy or any other infectious disease are considered by law as having no commercial value.

The carcass must be destroyed by burning, if practicable; otherwise buried under four feet of earth.

Quarantine may not be released in any case until the owner has disinfected and otherwise cleaned up as directed by health-officers.

Health-officers must realize that the law imposes important duties upon them, and severe penalty for either neglect or refusal to perform their duties. They should impress the fact upon the owners that the law also imposes duties and penalties upon them.

Glanders-farcy is very fatal and easily communicable to persons, if the matter from the nose or farcy-sores finds lodgment in any cut or scratch upon the hands or face, or if the matter gets into the nose or eyes.

The State Board of Health employs a field-veterinarian who is almost constantly on the road attending to unusually serious outbreaks of infectious diseases among domestic animals; to outbreaks where there is difference of opinion between veterinarians who have been called into the case, and to places where there are no competent veterinarians.

### TUBERCULOSIS.

This subject has been quite fully reported in my several quarterly reports during the past year, and need not be enlarged upon at this time. I am still satisfied that, for the present, the most practical plan for dealing with tuberculosis is to do our utmost to encourage owners to have their cattle tested, and to insist that herds be tested with tuberculin where there is good reason to suppose that this disease is present. I believe that it is not practical at present to attempt any sweeping measures of eradication by exterminating tuberculous cattle. We have continued to furnish the tuberculin freely, together with full instructions concerning its use, to and through local health-officers.

*Conference with health-officers of St. Paul and Minneapolis.* As reported at a previous meeting of this board the committee on infectious diseases of animals had two meetings with the health departments of the two cities, at which meeting certain important matters were agreed upon. Representatives of the three bodies agreed that a public abattoir for each city was an important thing, for which we must all work, and which must be secured before the meat-supplies of the two cities can be properly inspected. We also agreed concerning the tuberculin-test, that cattle which react upon the tuberculin-test ought to be permanently branded; that the brand ought to be uniform, and that the most practical plan is to brand with a hot iron in a conspicuous place. We agreed to use a small capital "T" and brand upon the left hip, brand to be furnished by the State Board.

It was also decided that the Government regulations concerning inspection of tuberculous cattle that are slaughtered for food-purposes should be adopted, the essential point being that cattle may be affected with tuberculosis and yet fit for human food, and that the only way to estimate upon this point is by competent inspection at the time of slaughter. The health department of each city has agreed to send duplicates of their tuberculin- and mallein-records for proper filing with this department of the State Board.

### Cornstalk Disease.

Cornstalk disease is the very unscientific, but very expressive name for a disease that has prevailed in many portions of the State during the fall months. This trouble has been reported from the following counties: Benton, Nicollet, Brown, Blue Earth, Sherburne, Houston, Jackson, Chisago, Wright, Freeborn, Martin, Redwood; in all, twelve counties.

The disease is probably not a new one, but has only been recognized and described within a few years. There is no evidence that the disease is infectious, so it does not come within the jurisdiction of the State Board of Health, and yet it is invariably reported as an infectious disease, and has caused a great deal of alarm. The etiology of this disease has not been worked out as yet. We know, however, that it usually appears among cattle that have been turned out in the cornstalk-fields; that it rarely appears among cattle that are fed shocked corn, and that the cattle die in comparatively large numbers and very suddenly. The attached circular has been sent out quite freely by the Experiment Station and State Board of Health to local papers in infected portions of the State, and also sent out freely in correspondence with the chairmen of town boards in southern portions of the State :

### *Cornstalk Disease.*

A number of reports have been coming in from various parts of the State to the effect that this disease has appeared among cattle that have been turned out in the stalks, and this is written as a matter of warning for farmers who wish to feed their cornstalks to cattle in the field. It is scarcely necessary to suggest that the cattle should be taken out of the field wherein the disease appears, as early as possible, and not put back in the same field for at least a month, and then very cautiously.

*Symptoms.* This disease appears very suddenly. The diseased animals may be seen standing apart from the others, and showing a peculiar humped-up appearance, the tail switching, eyes with a wild expression, and sometimes cattle are actually delirious. As the disease becomes more pronounced the symptoms of pain and delirium increase. Death frequently results within twenty-four hours after the first symptom is noticed. Cattle affected with this disease are frequently reported as rabid, and have been the cause of considerable excitement in a neighborhood, the people thinking it rabies and that the cattle have been bitten by a mad dog. A mad-dog story usually becomes attached sooner or later to the history of the outbreak. The disease is probably not contagious, and many cases appear in close succession, simply because the cattle have been all living under similar conditions and eating the same feed.

*Prevention.* The prevention of this disease is very simple. It appears among cattle that have been turned out in the cornstalk-field, and the cattle usually die while in the field. If the cattle which have been dying have been kept in the stalks and the symptoms correspond with those I have given, they should be taken out as soon as possible from the fields wherein the disease has appeared, and be induced to take as much salt and water during the next few days as possible. The disease does not seem to appear when the corn has been cut and shocked and the cattle are fed this shocked corn. The trouble comes most frequently to cattle that are turned in the stalks hungry, and for this reason it is safer to turn cattle in for the first time after a generous feed of what they have been accustomed to, and let them remain in the field. The disease appears most frequently among cattle that are taken out at night, put back in the morning, etc.

*Treatment.* Cases die so suddenly, and the disease is so easily checked without medicines, that it does not seem worth while to suggest treatment.

M. H. Reynolds.

### Miscellaneous Diseases.

During the year there have been occasional outbreaks of black-leg among young cattle in western portions of the State.

Anthrax has been reported, but the evidence is imperfect.

Scab in sheep does not prevail to a serious extent in this State, but there is imminent danger that we may have much trouble in the future from the large shipments of sheep that come every fall from other States where the disease is prevalent. The matter can only be controlled by the coöperation of interested States, with the assistance of the railroads and General Government. In his twelfth and thirteenth annual reports, Dr. Salmon, chief of the Bureau of Animal Industry, made certain recommendations concerning this matter which ought to be seriously considered by those who are vitally interested.

There have been a number of cases of canine rabies in the State, mainly in the vicinity of St. Paul, and one outbreak of bovine rabies which resulted in the loss of four cattle. This occurred in Wright County.

One serious outbreak of so-called verminous bronchitis at Taopi, in Mower County, has been investigated. This outbreak appeared in the fall among the spring calves. Ten had died at the time of the first report; others died later, and the loss in thrift and growth was quite serious. Verminous bronchitis is certainly an unsatisfactory name for this disease, for it is not distinctly a bronchitis, and the disease appears under conditions that give a strong suspicion that the strongyles are not the primary cause of the disease.

### In Relation to Experiment Station and Farmers' Institutes.

I have been greatly pleased with the way in which the veterinary work in connection with the State Board of Health has harmonized with the veterinary division of the University of Minnesota Experiment Station. As Experiment Station veterinarian I have charge of matters concerning non-infectious diseases of domestic animals, and from the State Board of Health comes the section of infectious diseases, and the two have fitted together very nicely.

The relation that has existed between the State Farmers' Institute and the veterinary portion of the State Board of Health work has been very harmonious and very helpful.

### Field-veterinarian.

Concerning the work of Dr. S. D. Brimhall, as field-veterinarian to the State Board of Health, I will only take time to say that his work has been very satisfactory both to the farmers and stockmen with whom he has to deal, and to me as director of the veterinary department, and, I trust, to Dr. Bracken as executive officer of the Board. Although his work necessarily brings him at times into something of a conflict with owners, I am pleased to report that in every case, except one, he has been able to obtain their full confidence. He seems to have a happy way of dealing with people in this kind of work. I believe that in attending to cases of unusual severity, to matters over which there is dispute by different veterinarians or health-officers, and by going to places where there are no competent veterinarians, he is doing good work for the State.

### Proposed Circular: General Suggestions for Local Health-officers.

Township supervisors should bear in mind that under the law they are local health-officers, and, at their meetings, should discuss the health of farm-stock in their township, just as they discuss the condition of the roads or infectious diseases among people.

If any suspicious disease has appeared among domestic animals, it should be reported at the meeting by the member who knows of it.

It is the duty of the local health-officers to take the initiative in all cases of infectious diseases. They should not wait for complaints from neighbors or for instructions from the State Board.

Local health-officers should make it their rule to act on their best judgment, and not be influenced unduly by what owners and neighbors may say or think.

The law specifies that local health-officers shall report within twenty-four hours to the State Board after they have information concerning any infectious disease among stock.

Local health-officers should keep a complete file of all blanks and circulars which they may need for infectious diseases among domestic animals, and should distribute circulars in the neighborhood where they will do most good. Local health-officers should impress upon owners that quarantine and other regulations which local boards may make are supported by law and must not be violated, and that such violation is punishable by severe penalty.

As a general rule, the township pays all the expenses which may be incurred by the town board in pursuance of this work, and incidental travelling expenses such as hotel and livery bills which may be incurred by the field-veterinarian or other representative of the State Board while doing work for that township.

*Lumpy jaw.* All cattle affected with the disease known as lumpy-jaw should be quarantined, and when killed should be inspected by one of the local board. If all the internal organs are healthy the meat may be used or sold for food, but if any of the internal organs are diseased the meat shall be condemned.

*Tuberculosis.* All the cattle which show symptoms of tuberculosis should be quarantined at once and the entire herd tested with tuberculin. All that react should be kept in quarantine and none of their products used as food for man or animals. If the owner desires, they may be killed at once, or tested again not sooner than two months and not later than three months, as provided by law. If they give distinct reactions on second test, they should be killed and the stable cleaned and disinfected, same as for glanders.

